# Correlation between Obstructive Sleep Apnea Syndrome and Cardiac Disease Severity

**DOI:** 10.1155/2014/631380

**Published:** 2014-02-16

**Authors:** Hamid Reza Javadi, Shabnam Jalilolghadr, Zohreh Yazdi, Zeinab Rezaie Majd

**Affiliations:** ^1^Qazvin University of Medical Sciences, Qazvin, Iran; ^2^Metabolic Disease Research Center, Qazvin University of Medical Sciences, Qazvin, Iran; ^3^Social Determinants of Health Research Center, Qazvin University of Medical Sciences, Qazvin 3413689414, Iran

## Abstract

*Background*. Obstructive sleep apnea (OSA) syndrome is one of the most common respiratory disorders in humans. There is emerging evidence linking OSA to vascular disease, particularly hypertension. The underlying pathophysiological mechanisms that link OSA to cardiovascular diseases such as hypertension, congestive heart failure, and atrial fibrillation are not entirely understood. The aim of this study was to investigate the association of obstructive sleep apnea hypopnea syndrome (OSAHS) with coronary atherosclerotic disease (CAD). *Methods*. A questionnaire survey based on Berlin questionnaire and Epworth Sleepiness Scale (ESS) was conducted among 406 patients to assess demographic data and the symptoms, such as excessive daytime sleepiness and snoring. Epworth Sleepiness Scale and Berlin questionnaire were completed by all of the patients. Venous blood samples were obtained for biochemical tests. Characteristics of coronary arteries were collected from angiographies' reports. All patients were divided into two groups based on results from Berlin questionnaire: low risk patients for OSA and high risk patients for OSA. Data were analyzed by SPSS software version 13. *Results*. Mean age of patients was 61.8 ± 10.5. 212 (52.2%) patients were categorized as high risk group for apnea. Also, excessive daytime sleepiness was reported in 186 patients (45.8%). The severity of coronary artery involvement, daytime sleepiness, and electrocardiogram abnormalities was significantly higher in high risk patients for OSA compared with low risk patients. High risk patients had higher level of FBS and LDL and lower level of HDL cholesterol (P < 0.05). *Conclusion*. Our study found a strong correlation between the number of stenotic vessels and OSA. Based on our findings, OSA can be a predisposing factor for cardiac diseases.

## 1. Introduction

Obstructive sleep apnea syndrome (OSAS) is a common disorder. It is estimated that 4% of males and 2% of females suffer from this disorder [1]. The clinical findings associated with sleep apnea are habitual snoring and disturbance in sleep quality and daily cognitive dysfunction. The respiratory disorders have not only effect on sleep quality but also have destructive effects on the quality of waking hours. Excessive sleepiness during the day time is one of the main signs of sleep apnea syndrome [2–4]. 

Different studies showed that obstructive sleep apnea (OSA) may be a risk factor for incidence of some medical conditions such as diabetes type 2 and headache. Cardiovascular diseases are a group of disorders which have causal relationship with sleep apnea. According to surveys, the incidence of OSA in patients with cardiovascular diseases is 30 to 50 percent [5, 6]. The correlation between some cardiovascular diseases with sleep apnea is investigated. These are hypertension, myocardial infarction, arrhythmia, cardiomyopathies, heart failure, and cardiac attack [7–9].

The standard method for diagnosis of OSA is overnight polysomnography (PSG). PSG included electroencephalogram, electromyogram, electrocardiogram (ECG), oxymetery, oronasal air flow, and respiratory efforts. These parameters can determine existence and intensity of sleep apnea. Using standard PSG for screening purpose is impossible, because of high cost and time consumption of this procedure. There are also some questionnaires that assess the probability of sleep apnea occurrence. Among them, Berlin questionnaire and Epworth Sleepiness Scale (ESS) are the most common methods used for sleep apnea screening in several studies [10].

ESS is a method for evaluation of excessive drowsiness during the day. However, this is a subjective method, nonexpensive, easy to do, and available [11].

A survey on patients with OSA in 20 teaching hospitals showed that results of ESS compared with PSG findings for assessing apnea. Sensitivity and specificity of Berlin questionnaire for assessing OSA are 85% and 77%, respectively [12, 13].

According to findings of above-mentioned studies, this study is designed to assess the prevalence of OSA and its relation to angiography's results in patients with cardiovascular diseases.

## 2. Material and Methods

Subjects were patients (male or female) aged less than 70 years old who were scheduled for coronary angiography. In this prospective study, all patients were recruited at the Heart Clinic of Boali-Sina University Hospital from May to October 2009. Indications for cardiac catheterization were history of chest pain or pathologic findings in exercise ECG. A total of 845 patients were recruited for the study, and 406 of them had criteria for doing angiography test.

Both right and left coronary angiograms were performed in all patients. Coronary artery stenosis was considered significant if there was ≥70% diameter stenosis.

Patients with history of cerebrovascular attack (CVA), COPD, and heart failure and who took sedative drugs were excluded from study. Patients with oxygen dependency and coronary disorders with ejection fraction less than 40% were excluded too. The study protocol was explained for all patients and written informed consent was obtained from all subjects.

Height, weight, and waist and neck circumferences were measured for all subjects. Body mass index (BMI) was calculated by dividing weight (Kilogram) on squared height (Meter). Blood pressure also was measured with mercury barometer.

Demographic characteristics were collected using a questionnaire including age and sex. ESS and Berlin questionnaires were filled out for all subjects. The ESS is an eight-item questionnaire that is a simple and inexpensive instrument to measure subjective daytime sleepiness. The cutoff point for excessive daytime sleepiness is considered to be equal or greater than 10 [12]. Berlin questionnaire has three categories. The first category has five questions about snoring. Second category with four questions is about daytime somnolence. The last category with 1 question asks about high blood pressure. High risk for OSA is considered if two or more categories are positive [13].

Venous blood samples obtained for biochemical tests including LDL cholesterol, HDL cholesterol, and fasting blood sugar (FBS). ECG characteristics (like heart rhythm, rate, ischemic changes, and ST elevation and necrosis signs) were noted.

All data were processed by SPSS software version 13. Data are shown as the mean ± standard deviation or number (percentage). Differences between groups were analyzed by Student *t*-test, Chi-squared test, and one-way analysis of variance. Logistic regression was used to calculate the odds ratio (OR) and confidence interval of variables, while adjusting for possible confounders. Predictive value (*P* value) less than 0.05 was considered significant.

## 3. Results

Subjects mean age was 61.83 ± 10.5 years old (ranged from 40 to 70 years old). Mean value of BMI and neck circumference were 26.7 ± 3.6 Kg/m^2^ and 37.4 ± 1.4 cm, respectively.

Among subjects, 212 (52.2%) were categorized as high risk group for apnea and 194 (47.8%) as low risk group. Findings of ESS questionnaire were as follows: 220 (54.2%) were without sleepiness (ESS < 10) and excessive daytime sleepiness was reported in 186 (45.8%).

Studied parameters are shown in Tables 1 and 2.  Table 3 compares Berlin questionnaire, ESS, and other CV risk factors in patients with different severity of coronary artery disease (1-, 2-, and 3-vessel disease of Gensini score).

Table 4 shows the results of the logistic regression analysis on the associated factors of the presence of high risk of obstructive sleep apnea. The variables included in the univariate and multivariate regression model were age, sex, BMI, smoking, neck circumference, waist circumference, triglyceride, HDL cholesterol, FBS, daytime sleepiness, and number of vessels with stenosis in angiography. As the table shows, gender and BMI were not statistically associated with the presence of OSA. The variables with the highest ORs related to the presence of OSA were sex, FBS, daytime sleepiness, and number of vessels with stenosis in angiography.

## 4. Discussion

In the present study, high prevalence of OSA was reported in patients scheduled for angiography. Some studies showed that sleep breathing disorders independent of other risk factors can be an important risk factor for cardiovascular diseases. On the other hand, sleep breathing disorders can increase incidence of cardiovascular diseases more than two- to threefold compared with population without it [1–4]. Thus, OSA coexists in 30 to 50 percent of patients with coronary heart diseases [5].

Our findings also demonstrated that the intensity of coronary artery disease is higher in patients with OSA compared with the others. In the present study, patients with 2 to 3 vessels stenosis were classified in high risk group for apnea (according to Berlin questionnaire), while patients with single vessel disease had low risk for OSA. These findings are consistent with data from other studies. The study conducted by American Sleep Research Center showed that risk of cardiovascular events in patients with Apnea-Hypopnea Index (AHI) more than 5/hour is higher than those without apnea [14]. In the other studies, authors revealed that subjects with AHI more than 20/hour have higher risk of myocardial infarction, angina, and coronary events [15, 16].

The mechanism of this correlation is not well known, but the evidences imply that breathing disorders during sleep can result in high systolic blood pressure. Other studies showed that OSA can also result in increased fibrinogen and C-reactive protein levels. These substances can be the initial step of thrombosis process in coronary arteries [17].

In present study, OSA signs were common in patients and were correlated with coronary stenosis. We evaluated daytime sleepiness by using of ESS questionnaire. There was a significant correlation between excessive sleepiness during day (as a sign of OSA) and intensity of OSA. Parish and Somers studied 175 patients with coronary artery disease and demonstrated that 56% of them suffered from snoring [18]. Other findings from present study showed that 39% of patients with single coronary artery stenosis had high risk of having OSA screened by Berlin questionnaire, while 60 and 62 percent of patients with 2 or 3 vessels stenosis were stratified in high risk group for OSA.

The relation between OSA and coronary artery disease was studied frequently. Young et al. reported that the percentage of two or three vessels disease was only 12% in patients without OSA, but 80% of patients with OSA have stenosis in at least two coronary vessels [1]. These evidences imply the significant correlation between OSA and coronary artery disease.

We also studied the relation between ECG characteristics and parameters filled out in Berlin questionnaire. 60% of patients with ischemic evidence in ECG and 81% of patients who had pathologic Q in their ECG were also categorized in the group with high risk for OSA. Mooe et al. showed that 30% of patients who had ST-depression in their ECG were in high risk group for OSA [4].

We detected the lower level of HDL cholesterol and higher level of triglyceride in patients with high risk of OSA compared with patients with low risk of OSA. Pang and Terris revealed the significant correlation between hypercholesterolemia (specially the LDL cholesterol level) with heart diseases [10]. The findings of another study in German population showed that continuous positive airway pressure (CPAP) can increase HDL cholesterol level and decline the LDL cholesterol level in patients with OSA [19].

There were some limitations in our study. First of all we could not use polysomnography for diagnosis of apnea. We did not use PSG for our participants, owing to high cost and inconvenience for hospitalized patients. Secondly, we could not control the effect of all CAD risk factors. That was because of multiplicity of risk factors associated with coronary artery disease.

Based on our findings, OSA can be a predisposing factor for cardiac diseases. It seems that screening of OSA is beneficial in patients with cardiac diseases or even patients with hypertriglyceridemia or patients with low level of HDL cholesterol which does not respond to therapy. It can lead to early treatment and prevent the high morbidity and mortality rate of heart diseases. It can be also cost-beneficial, because it decreases the rate of coronary bypass and hospitalization.

## Figures and Tables

**Table 1:** Demographic characteristics and clinical findings in patients with or without OSA.

	Low risk patients *N* = 194	High risk patients *N* = 212	*P* value
Age	61.4 ± 10.6	63.8 ± 9.3	0.09
Gender			
Male	61 (15%)	174 (42.8%)	0.041
Female	133 (32.7%)	38 (9.3%)	
BMI	25.7 ± 3.4	27.6 ± 3.7	0.039
Smoking			
Yes	64 (15.7%)	78 (19.2%)	0.076
No	130 (32%)	134 (33%)	
Neck circumference	36.9 ± 1.5 (30.3–39.7)	37.6 ± 1.4 (33.6–42.4)	0.021
Waist circumference	91.8 ± 8.3 (83–95)	93.6 ± 8.9 (84–98)	0.034

*Data are number (percentage) and mean ± standard deviation (minimum–maximum).

**Table 2:** Comparing other risk factors in patients with or without OSA.

	Low risk patients *N* = 194	High risk patients *N* = 212	*P* value
Triglyceride (mmol/L)	137.1 ± 9.2	183.5 ± 14.9	0.004
HDL (mmol/L)	31.2 ± 5.2	27.8 ± 6.1	0.018
Fasting blood sugar (mg/dL)	107.5 ± 9.3	126.8 ± 6.7	0.001
Blood pressure (sys)	118.8 ± 20	132.2 ± 15.5	0.024
ESS	10.3 ± 4.7	13.8 ± 4.8	0.007
Number of vessels			
One vessel	118 (60.2%)	78 (39.8%)	0.005
Two vessels	61 (35.9%)	109 (64.1%)	
Three vessels	15 (37.5%)	25 (62.5%)	
EKG			
Ischemic	69 (17%)	99 (24.4%)	
Nonischemic	125 (30.8%)	113 (27.8%)	0.004
Q-wave	24 (5.9%)	64 (15.8%)	
Non-Q-wave	170 (41.9%)	148 (36.4%)	0.002
ST elevation	25 (5.7%)	97 (23.9%)	
Non-ST elevation	169 (42.1%)	115 (28.3%)	0.004
ST depression	79 (19.4%)	92 (22.7%)	
Non-ST depression	119 (29.3%)	116 (28.6%)	0.083

*Data are presented as number (percentage) and mean ± standard deviation.

**Table 3:** Comparing Berlin questioner, ESS, and other CV risk factors with coronary artery disease severity (1-, 2-, and 3-vessel disease of Gensini score).

	Number of vessels with stenosis			*P* value
	One vessel *N* = 196	Two vessels *N* = 170	Three vessels *N* = 40	
ESS	9.2 ± 2.7	11.7 ± 3.1	12.2 ± 1.6	<0.001
Patients with OSA	95 (23.4%)	53 (13%)	64 (15.6%)	0.002
Triglyceride	145.7 ± 12.3	137 ± 9.3	178 ± 7.9	0.03
HDL	37.2 ± 7.2	26.9 ± 4.9	29.1 ± 6.1	0.009
Fasting blood sugar	109.8 ± 8.9	129.8 ± 9.4	136.4 ± 14.1	0.03
Blood pressure (sys)	115.8 ± 6.9	139.5 ± 9.3	145.8 ± 14.1	0.007

*Data are presented as mean ± SD and number (percentage).

**Table 4:** Univariate and multivariate logistic regression results on associated factors of the presence of OSA.

	High risk of OSA *N* (%)	Univariate relative risk OR (95% CI)	*P* value	Multivariate relative risk OR (95% CI)	*P* value
Age	—	—	0.12	—	0.08
Gender					
Male	174 (42.8%)	1.3 (1.1–1.5)	0.003	1.25 (1.2–1.4)	0.001
Female	38 (9.3%)				
BMI	—	1.1 (1.08–1.12)	0.01	1.1 (1.9–1.11)	0.01
Smoking					
Yes	78 (19.2%)	—	0.21	—	0.076
No	134 (33%)				
Neck circumference	37.6 ± 1.4	2.1 (1.3–2.4)	0.02	2.1 (1.5–2.3)	0.004
Waist circumference	93.6 ± 8.9	1.4 (1.1–1.8)	0.01	1.4 (1.2–1.8)	0.002
TG (mmol/L) ≥150 mg/dL	316 (77.8%)	3.1 (2.4–4.2)	0.004	3.1 (2.5–4.1)	0.001
HDL (mmol/L) ≤40 mg/dL	288 (70.9%)	1.9 (1.4–2.8)	0.008	1.8 (1.4–2.7)	0.002
FBS >110	76 (18.7%)	2.7 (1.6–3.5)	0.02	2.8 (1.9–3.2)	0.006
Blood pressure (sys)	132.2 ± 15.5	2.1 (1.3–2.6)	0.005	2.3 (1.4–2.5)	0.003
